# Scalable metagenomics alignment research tool (SMART): a scalable, rapid, and complete search heuristic for the classification of metagenomic sequences from complex sequence populations

**DOI:** 10.1186/s12859-016-1159-6

**Published:** 2016-07-28

**Authors:** Aaron Y. Lee, Cecilia S. Lee, Russell N. Van Gelder

**Affiliations:** 1 Department of Ophthalmology, University of Washington School of Medicine, Box 359608, 325 Ninth Avenue, Seattle, WA 98104 USA; 2 Departments of Biological Structure and Pathology, University of Washington School of Medicine, Seattle, WA USA

## Abstract

**Background:**

Next generation sequencing technology has enabled characterization of metagenomics through massively parallel genomic DNA sequencing. The complexity and diversity of environmental samples such as the human gut microflora, combined with the sustained exponential growth in sequencing capacity, has led to the challenge of identifying microbial organisms by DNA sequence. We sought to validate a Scalable Metagenomics Alignment Research Tool (SMART), a novel searching heuristic for shotgun metagenomics sequencing results.

**Results:**

After retrieving all genomic DNA sequences from the NCBI GenBank, over 1 × 10^11^ base pairs of 3.3 × 10^6^ sequences from 9.25 × 10^5^ species were indexed using 4 base pair hashtable shards. A MapReduce searching strategy was used to distribute the search workload in a computing cluster environment. In addition, a one base pair permutation algorithm was used to account for single nucleotide polymorphisms and sequencing errors. Simulated datasets used to evaluate Kraken, a similar metagenomics classification tool, were used to measure and compare precision and accuracy. Finally using a same set of training sequences we compared Kraken, CLARK, and SMART within the same computing environment. Utilizing 12 computational nodes, we completed the classification of all datasets in under 10 min each using exact matching with an average throughput of over 1.95 × 10^6^ reads classified per minute. With permutation matching, we achieved sensitivity greater than 83 % and precision greater than 94 % with simulated datasets at the species classification level. We demonstrated the application of this technique applied to conjunctival and gut microbiome metagenomics sequencing results. In our head to head comparison, SMART and CLARK had similar accuracy gains over Kraken at the species classification level, but SMART required approximately half the amount of RAM of CLARK.

**Conclusions:**

SMART is the first scalable, efficient, and rapid metagenomics classification algorithm capable of matching against all the species and sequences present in the NCBI GenBank and allows for a single step classification of microorganisms as well as large plant, mammalian, or invertebrate genomes from which the metagenomic sample may have been derived.

## Background

Next generation sequencing technology has enabled characterization of metagenomics through massively parallel genomic DNA sequencing. The complexity and diversity of environmental samples such as the human gut microflora, combined with the sustained exponential growth in sequencing capacity, has led to the challenge of identifying microbial organisms by DNA sequence [1, 2]. The library of sequenced DNA fragments mapped to an identified taxonomy species has been growing in parallel; the latest release of NCBI Genbank (v209) has catalogued 1.99 × 10^11^ basepairs of cDNA and genomic DNA from 1.87 × 10^8^ records [3] (http://www.ncbi.nlm.nih.gov/news/08-19-2015-genbank-release-209/). The computational challenge has been to rapidly and accurately identify species level DNA sequences from next generation metagenomic shotgun sequencing data.

Currently the most widely used classification algorithm, BLAST [4], relies on indexing unique fragments of DNA that narrow the search space. While BLAST works well for small numbers of sequences, the algorithm scales poorly to the large number of reads generated by next generation sequencing files [5]. Other sequence alignment software has been created specifically adapted to next generation sequencing output such as Bowtie2 [6], Burrows-Wheeler Aligner [7], and Short Oligonucleotide Analysis Package [8]. These alignment software work well for the precise alignment of a large number of next generation sequencing reads against single organism genomes but scale poorly when attempting to align reads against all known DNA sequences. MEGAN and MetaPhyler have been developed to work with BLAST specifically for the use of metagenomic sequencing classification [9, 10]. However even though these probabilistic approaches have high accuracy [11, 12], they remain limited by the computational expensive nature of BLAST. In addition, empirical approaches have also used machine learning algorithms with both supervised [13–16] and unsupervised methods [17–19].

Recently Kraken was developed to specifically address the problem of classifying next generation sequencing output from metagenomics projects [5]. Briefly, Kraken works by creating a k-mer database mapped to the lowest common ancestor, reducing the search space significantly. By doing so, Kraken performs exact k-mer matching and maps reads against its database with high speed and throughput. Validation of Kraken suggested processing of 4 million reads per minute at a rate over 900 times faster than MegaBlast [5]. The limitations of Kraken includes the long execution time and memory consumption during the database construction as well as the current databases being limited to bacterial, archaeal, and viral genomes, necessitating the elimination of host genomic DNA prior to classification using Kraken.

In addition to Kraken, a number of other approaches have been published. In particular CLARK [20] and LMAT [21] have been shown to have similar if not higher accuracy while maintaining the impressive throughput of Kraken. LMAT, similar to Kraken, attempts to utilize taxonomy information to reduce the database of k-mers, but current implementations are limited to microbial genomes and do not include mammalian sequences. CLARK attempts to decrease the k-mer search space by only indexing keys that uniquely identify a given taxonomy level and offers several modes of execution, including a version called CLARK-L that is optimized for limited RAM environments by subsampling the database to smaller fraction. All three techniques, Kraken, LMAT, and CLARK, attempt to limit the k-mer search space by either finding the least common ancestor (LCA) k-mers or finding discriminatory k-mers that uniquely identify an organism at a given taxonomy level.

Recently, the MapReduce programming model [22] has caused a substantial shift in the way that large data sets may be distributed in parallel within a computing cluster. For example, Google used the MapReduce [23] framework to regenerate their index of the Internet, and the MapReduce framework has become popularized as a generic framework to solve big data bioinformatics problems in many-core cluster systems [24–27]. Database sharding has been used in other fields to horizontally scale very large sets of data and can reduce the each subset of the database into a datastructure in memory limited environments [28, 29]. Unlike prior algorithms which limited the k-mer search space, we sought to leverage parallel computing and a MapReduce computational framework with a sharded database to create a scalable complete search heuristic for next generation sequencing files from metagenomics projects.

## Implementation

### Computational infrastructure

The University of Washington provides a shared high-performance computing cluster known as Hyak. Currently UW Hyak has 9,028 Intel Xeon processing cores with 834 computational nodes. Each node used to test computational scaling contained 16 CPU cores with 64 GB of memory.

### Construction of database

The v209 release of NCBI Genbank was downloaded (September 2015) and each Genbank accession was linked using the NCBI Taxonomy database to a single species and class. Using parallelization across 156 cores and a MapReduce framework, the genomic DNA was then virtually cut at every 30 basepairs, and each 30-mer was linked to the corresponding species and class and sorted. Finally merge sort was used to combine all the sorted 30-mers for classification. The dataset was then split into shards based on the first four basepairs of each 30mer creating a 256 separate databases that could be deterministically searched. The databases were saved in a hashtable format that could be loaded at runtime into memory by each search program.

### Description of search heuristic

**Fig. 1:**
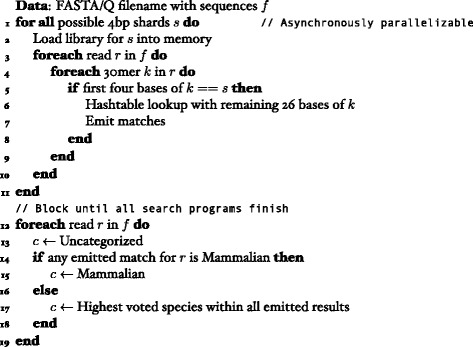
Sketch of search strategy in pseudocode

A total of 256 search programs are started asynchronously in parallel with each program assigned a 4 basepair shard as part of the mapping step. Each search program then iterates through the list of sequences in FASTA or FASTQ format and slides a 30 basepair window if the first 4 basepairs match the assigned shard definition of the executing program. The remaining 26 basepairs are then used to execute an in-memory hash-table lookup (Fig. 1). The reverse complement is also checked for every read. Each successful match to a species, genus, or class is kept and recorded. In addition, a 1-edit distance permutation algorithm was created to generate every possible one base-pair substitution permutation of the 30-mer search to account for sequencing errors and single nucleotide polymorphisms, without accounting for insertions or deletions. The results of each program are sequentially reduced to create the final classification results. Matching is performed at the species level and multiple matches against different organisms are collected. If any match is mammalian then the read is classified as mammalian; the highest voted match at the species, genus, and class taxonomy levels are calculated for each read for the final classification. If the highest classification for a read is a tie, then the read is labeled as ambiguous for a given taxonomy level.

### Datasets tested

Simulated datasets (HiSeq, MiSeq, and simBA5) were taken from the publicly available datasets that were used to evaluate Kraken [5].

In a previous clinical trial of acute conjunctivitis/epidemic keratoconjunctivitis (NV-422 Phase IIB/III, NovaBay, clinicaltrials.gov: NCT01532336), a total 500 patients with clinical signs and symptoms of epidemic keratoconjunctivitis were recruited worldwide. Institutional review board approval was obtained through Goodwyn IRB (Cincinnati, OH, approval number: CL1104) Clinical research adhered to the tenets of the Declaration of Helsinki and was conducted in accordance with Health Insurance Portability and Accountability Act regulations. Written informed consent was obtained before participation for all participants in the study. Conjunctival samples from the upper/lower tarsal conjunctiva and fornix were collected using sterile dry swabs (Copan diagnostics inc., Murrieta, CA). Genomic DNA was isolated from conjunctival swabs using Qiagen Blood & Tissue DNA Kit (Qiagen, Inc., Venlo, the Netherlands) as per protocol. Three samples were randomly selected for whole genome sequencing (WGS). One nanogram of genomic DNA from each sample was used to prepare libraries for WGS according to the manufacturer’s instruction using Illumina Nextera XT Sample Prep Kit (Illumina, Inc, San Diego, CA). The DNA libraries were sequenced using MiSeq System following the manufacturer’s standard protocols (Illumina, Inc, San Diego, CA). Three conjunctival samples were used from this clinical trial collected from patients on the day of enrollment prior to the initiation of either placebo or the investigative drug. The FASTQ files for these samples have been uploaded to the NCBI SRA archive (SRR3033169, SRR3033245, and SRR3033274). Flash was used to preprocess the paired end libraries and Sickle was used for quality trimming [30].

In addition, data from the Human Microbiome Project [31] was downloaded as an additional metagenomic dataset. Specifically, three gut microbiome datasets (SRS019120, SRS014468 and SRS015055) were downloaded from the NCBI Sequence Read Archive, and Sickle again was applied prior to analysis of the samples.

### Evaluation of accuracy and speed

To allow for direct comparison of performance statistics, the same definition of sensitivity and precision were used as described by Wood et al. [5]. Briefly sensitivity was defined as the number of correct classifications of reads divided by the total number in each dataset. Precision was defined as the number of correct classifications divided by the total number of reads attempted to be classified.

### Comparison of SMART to Kraken and CLARK

In order to compare the accuracy and performance of the three tools, DNA sequence files from all the bacterial, viral, and archaeal sections of RefSeq were downloaded. For Kraken, CLARK, and SMART, the same sequences were used to build a database in each tool respectively following the documentation provided. The simulated datasets were then analyzed by each tool on the same computational node (16 CPU cores with 64 GB of RAM) in the UW Hyak with multithreading enabled to the maximum number of CPUs. For Kraken, the database was preloaded into memory for maximal performance as suggested by the creators of Kraken for users with NFS filesystems. For CLARK, the standard mode (−m 1) was used to analyze the simulated files as the program failed to start with other modes due to the RAM limitation. In order to calculate throughput, each program was run sequentially three times and the lowest execution time was utilized to calculate throughput.

### Software and statistics

Custom software was written in C++ and Ruby. Statistics were performed using R (http://r-project.org). Conjunctival classification results from Kraken were obtained using Illumina BaseSpace and NCBI Blast was run with the database downloaded on November 2015. Software depends on Google SparseHash (https://github.com/sparsehash/sparsehash) and GNU parallel (http://www.gnu.org/software/parallel/). Software used to run SMART, prebuilt libraries, and training of custom libraries is available at a public repository (https://bitbucket.org/ayl/smart).

## Results

After transferring all genomic DNA reads from the latest release of the NCBI GenBank (version 209), a total of over 1 × 10^11^ bp of 3.34 × 10^9^ sequences from 9.26 × 10^5^ species of 1.49 × 10^3^ classes were indexed. The number of sequences indexed and the total number of uniquely identifying sequences from the 20 most abundantly represented classes and species are shown in Tables 1 and 2 respectively. Over 3.28 × 10^9^ sequences (98.3 %) and 3.32 × 10^9^ sequences (99.6 %) were uniquely identifying of a single species and class respectively. With a 4 basepair shards, 256 separate hashtables were created and indexed using a quadratic probing hashtable structure. The uncompressed sharded files used 137 GB of hard disk space to store, with each shard on average consuming 0.53GB of space. Total database construction was completed within 1.5 h and each thread consumed less than 1GB of memory.

**Table 1:** Twenty most abundantly represented classes by 30 basepair fragments in Genbank

	Total			Unique		
Class	Sequences	Base Pairs	%	Sequences	Base Pairs	%
Mammalia	1.13 × 10^9^	3.38 × 10^10^	33.59	1.12 × 10^9^	3.37 × 10^10^	33.76
Liliopsida	3.23 × 10^8^	9.68 × 10^9^	9.62	3.22 × 10^8^	9.66 × 10^9^	9.69
Chromadorea	1.67 × 10^8^	5.00 × 10^9^	4.97	1.66 × 10^8^	4.99 × 10^9^	5.01
Actinopteri	1.57 × 10^8^	4.72 × 10^9^	4.69	1.57 × 10^8^	4.71 × 10^9^	4.72
Gammaproteobacteria	1.54 ×10^8^	4.62 × 10^9^	4.59	1.52 × 10^8^	4.56 × 10^9^	4.57
Solanaceae	1.19 × 10^8^	3.57 ×10^9^	3.55	1.19 × 10^8^	3.56 × 10^9^	3.57
Trematoda	1.10 × 10^8^	3.30 × 10^9^	3.28	1.10 × 10^8^	3.29 × 10^9^	3.31
Cestoda	9.06 × 10^7^	2.72 × 10^9^	2.70	9.06 × 10^7^	2.72 × 10^9^	2.73
Fabaceae	8.95 × 10^7^	2.69 × 10^9^	2.67	8.93 × 10^7^	2.68 × 10^9^	2.69
Bacilli	7.66 × 10^7^	2.30 × 10^9^	2.28	7.59 × 10^7^	2.28 × 10^9^	2.28
Actinobacteria	6.66 × 10^7^	2.00 × 10^9^	1.99	6.60 × 10^7^	1.98 × 10^9^	1.99
Aves	6.39 × 10^7^	1.92 × 10^9^	1.91	6.37 × 10^7^	1.91 × 10^9^	1.92
Betaproteobacteria	4.97 × 10^7^	1.49 × 10^9^	1.48	4.94 × 10^7^	1.48 × 10^9^	1.49
Brassicaceae	4.78 × 10^7^	1.43 × 10^9^	1.42	4.75 × 10^7^	1.43 × 10^9^	1.43
Insecta	4.66 × 10^7^	1.40 × 10^9^	1.39	4.64 × 10^7^	1.39 × 10^9^	1.40
Alphaproteobacteria	4.54 × 10^7^	1.36 × 10^9^	1.35	4.48 × 10^7^	1.34 × 10^9^	1.35
Echinoidea	3.79 × 10^7^	1.14 × 10^9^	1.13	3.77 × 10^7^	1.13 × 10^9^	1.13
Saccharomycetes	2.99 × 10^7^	8.98 × 10^8^	0.89	2.95 × 10^7^	8.86 × 10^8^	0.89
Clostridia	2.34 × 10^7^	7.02 × 10^8^	0.70	2.29 × 10^7^	6.87 × 10^8^	0.69
Vitaceae	2.24 × 10^7^	6.72 × 10^8^	0.67	2.23 × 10^7^	6.70 × 10^8^	0.67

**Table 2:** Twenty most abundantly represented species by 30 basepair fragments in Genbank

	Total			Unique		
Species	Sequences	Base Pairs	%	Sequences	Base Pairs	%
*Homo sapiens*	2.76 × 10^8^	8.29 × 10^9^	7.99	2.67 × 10^8^	8.00 × 10^9^	8.13
*Mus musculus*	2.03 × 10^8^	6.10 × 10^9^	5.88	2.02 × 10^8^	6.05 × 10^9^	6.14
*Rattus norvegicus*	1.55 × 10^8^	4.66 × 10^9^	4.49	1.54 × 10^8^	4.62 × 10^9^	4.69
*Bos Taurus*	1.26 × 10^8^	3.78 × 10^9^	3.65	1.24 × 10^8^	3.72 × 10^9^	3.78
*Sus scrofa*	1.23 × 10^8^	3.70 × 10^9^	3.56	1.23 × 10^8^	3.68 × 10^9^	3.74
*Zea mays*	1.02 × 10^8^	3.07 × 10^9^	2.96	1.02 × 10^8^	3.06 × 10^9^	3.11
*Danio rerio*	6.56 × 10^7^	1.97 × 10^9^	1.90	6.54 × 10^7^	1.96 × 10^9^	1.99
*Hordeum vulgare*	6.45 × 10^7^	1.93 × 10^9^	1.86	6.40 × 10^7^	1.92 × 10^9^	1.95
*Ovis canadensis*	5.80 × 10^7^	1.74 × 10^9^	1.68	5.62 × 10^7^	1.68 × 10^9^	1.71
*Cyprinus carpio*	5.50 × 10^7^	1.65 × 10^9^	1.59	5.49 × 10^7^	1.65 × 10^9^	1.67
*Solanum lycopersicum*	4.59 × 10^7^	1.38 × 10^9^	1.33	4.47 × 10^7^	1.34 × 10^9^	1.36
*Apteryx australis*	4.51 × 10^7^	1.35 × 10^9^	1.30	4.50 × 10^7^	1.35 × 10^9^	1.37
*Strongylocentrotus purpuratus*	3.76 × 10^7^	1.13 × 10^9^	1.09	3.74 × 10^7^	1.12 × 10^9^	1.14
*Spirometra erinaceieuropaei*	3.57 × 10^7^	1.07 × 10^9^	1.03	3.56 × 10^7^	1.07 × 10^9^	1.09
*Pan troglodytes*	3.55 × 10^7^	1.07 × 10^9^	1.03	3.20 × 10^7^	9.59 × 10^8^	0.97
*Oryza sativa*	3.09 × 10^7^	9.26 × 10^8^	0.89	2.90 × 10^7^	8.71 × 10^8^	0.89
*Nicotiana tabacum*	3.06 × 10^7^	9.17 × 10^8^	0.88	3.05 × 10^7^	9.14 × 10^8^	0.93
*Solanum pennellii*	2.72 × 10^7^	8.16 × 10^8^	0.79	2.60 × 10^7^	7.80 × 10^8^	0.79
*Echinostoma caproni*	2.50 × 10^7^	7.50 × 10^8^	0.72	2.50 × 10^7^	7.50 × 10^8^	0.76
*Triticum aestivum*	2.33 × 10^7^	7.00 × 10^8^	0.67	2.27 × 10^7^	6.80 × 10^8^	0.69

**Fig. 2:**
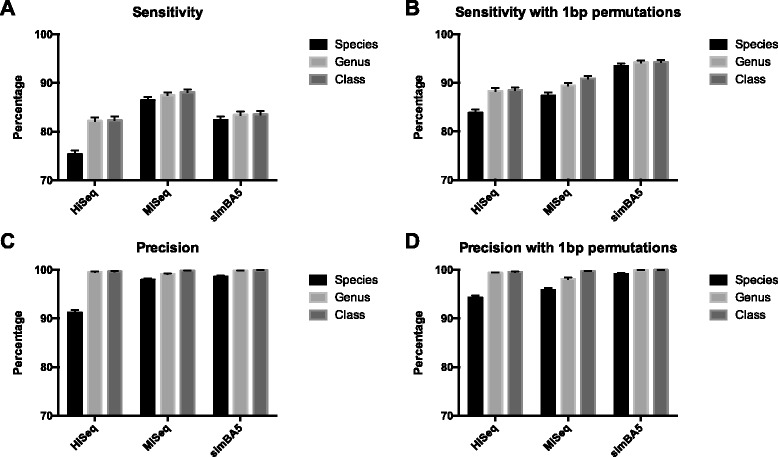
Accuracy results of deep search on simulated datasets using the Genbank library. **a** Sensitivity with exact matching at species, genus, and class levels for simulated datasets (HiSeq, MiSeq, and simBA5). **b** Sensitivity with 1 basepair permutations during search. **c** Precision with exact matching. **d** Precision with 1 basepair permutations during search. Error bars represent 95 % confidence intervals

**Fig. 3:**
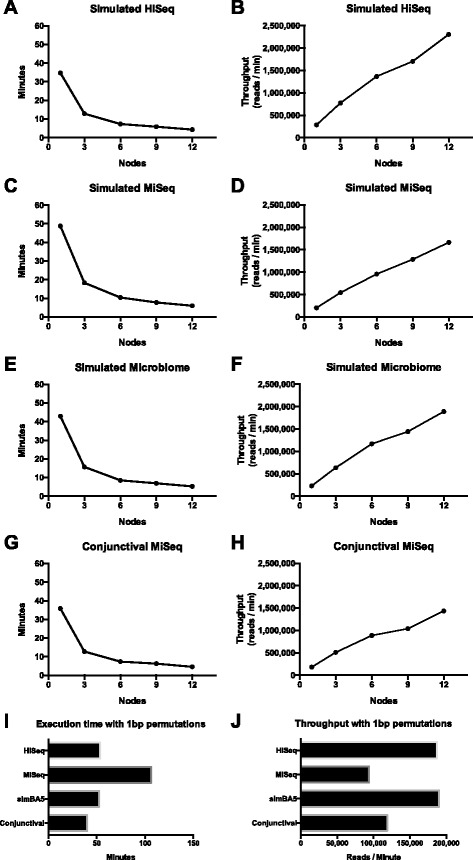
Computational scalability of SMART on a computing cluster using the Genbank library. **a**, **c**, **e**, **g** Overall execution time to complete processing of datasets with increasing number of computing nodes utilized. **b**, **d**, **f**, **h** Throughput measured in reads per minute processed with increasing number of computing nodes utilized. **i** Execution time of datasets with 12 nodes utilized and 1 basepair permutations during search. **j** Throughput of datasets with 12 nodes utilized and 1 basepair permutations during search

Using the same simulated datasets that were used to evaluate Kraken [5], we measured the sensitivity and precision at the species, genus, and class taxonomy levels (Fig. 2). On a single node with 12 search programs executing in parallel, each of the simulated datasets took a total of 30 min to finish. The maximum memory consumed by a single search program was 7.45 GB with an average of 3.78 GB used by each program. A 100 % utilization of each CPU core was noted during the execution of each search program. Using multiple nodes to further parallelize the computation, we achieved linear scaling in throughput with inversely proportional decreases in total computational time (Fig. 3). By increasing the number of nodes to 12, we achieved a maximum throughput of over 2.3 million reads per minute and the ability to classify each of the simulated datasets in under 5 min. Performance of classifying a “real-world” human conjunctival derived metagenomic next generation sequencing result did not show any difference in computational scaling (Fig. 3g and h). When the cost of 1 bp permutations was measured, there was on average a 12.17 times increase in execution time (Fig. 3i and j). However an average of 6.83 × 10^5^ additional reads (6.9 %) was classified in the three simulated datasets.

Because many metagenomic projects come from a single host organism, a major bioinformatic challenge is to effectively filter the host organism genomic DNA from the DNA of the microbial organisms. Indexing the totality of known DNA from the NCBI GenBank and using the NCBI taxonomy classes allows for simultaneous classification of all reads to both mammalian genomes and non-mammalian genomes without a need for a pre-filtering stage. To prevent false positive match for microbial DNA, a conservative approach was used in that if a read was classified even once as mammalian then it was considered to be mammalian in origin. Of note, in GenBank only 11.1 % and 0.4 % of all known 30-mers have perfect matches for bacterial and viral DNA, respectively, at the class taxonomy level.

**Fig. 4:**
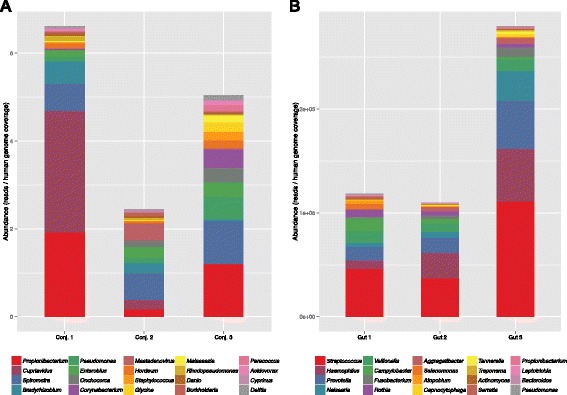
Twenty most common genera in metagenomics samples. **a** Human conjunctival metagenomics whole genome sequencing samples and **b** Human gut metagenomics whole genome sequencing samples with total reads normalized by coverage of human genome

Using this strategy, the whole genome sequencing results from three separate conjunctival samples and three gut microbiome samples from the Human Microbiome Project (SRS019120, SRS014468 and SRS015055) were analyzed with 1 basepair permutations (Table 3). In the human gut samples, on average 42.44 % of all reads were classified with 38.62 % matching non-mammalian DNA. On average, in the paucibacterial conjunctival samples 98.6 % of all the reads were classified; of these, 0.02 % matched non-mammalian DNA. The total reads by classified genus were normalized by the depth of coverage of the human genome in each sample to account for sequencing depth variability. The top twenty organisms from each sample are shown in Fig. 4.

**Table 3:** Classification results of metagenomics samples using SMART with the Genbank library

Sample	Total reads	Mammalian		Non-Mammalian				Unmatched	
				Ambiguous		Unique			
	*N*	*n*	%	*n*	%	*n*	%	*n*	%
Conjunctival 1	4,731,317	4,660,011	98.49	310	0.0066	1,627	0.034	69,369	1.50
Conjunctival 2	1,135,916	1,119,975	98.60	32	0.0028	173	0.015	15,736	1.38
Conjunctival 3	4,540,162	4,483,966	98.76	338	0.0074	1,332	0.029	54,476	1.20
Gut 1	2,439,314	102,982	4.22	176,810	7.25	661,399	27.11	1,498,123	61.42
Gut 2	760,562	37,461	4.93	65,144	8.57	218,409	28.72	439,548	57.79
Gut 3	2,326,530	54,012	2.32	160,774	6.91	868,078	37.31	1,243,666	53.46

To compare the three methods, one conjunctival sample was analyzed. Human reads were filtered using Illumina Basespace, and was run through Kraken and CLARK with libraries built using all the bacterial, viral, and archaeal sequences from RefSeq. Kraken attempted to classify 6.4 × 10^5^ non-human reads but 98 % were unable to be identified. Comparison of the same read results with SMART revealed that 83 % of unclassified reads by Kraken were mammalian DNA in origin. In addition, 69.8 % of microbial classified reads by Kraken also matched mammalian DNA by SMART. A comparison of the microbial matched reads by Kraken against BLAST revealed a similar trend (Table 4). In addition, a similar comparison was made with the results from CLARK; the majority of the reads classified by CLARK as microbial were identified by SMART as having mammalian origin and this was confirmed independently using BLAST (Table 5).

**Table 4:** Comparison of Kraken results to SMART using the Genbank library and BLAST for Conjunctival Sample 1

Kraken Genus	Kraken reads	SMART				BLAST			
		Mammalian	Same Genus	Other Genus	Unknown	Mammalian	Same Genus	Other Genus	Unknown
Top 10 classified									
*Altermonas*	1,545	1,249	0	283	13	1,227	45	238	35
*Propionibacterium*	323	90	232	0	1	8	314	0	1
*Mycoplasma*	100	100	0	0	0	77	0	0	23
*Pseudomonas*	93	5	86	0	2	6	85	0	2
*Pandoravirus dulcis*	88	88	0	0	0	44	0	0	44
*Pandoravirus salinus*	46	46	0	0	0	11	0	0	35
*Staphylococcus*	46	4	42	0	0	0	46	0	0
*Human Endogenous* *Retrovirus K113*	31	30	0	0	1	30	0	0	1
*Delftia*	30	7	23	0	0	2	28	0	0
*Corynebacterium*	28	3	24	0	1	0	28	0	0

**Table 5:** Comparison of CLARK results to SMART using the Genbank library and BLAST for Conjunctival Sample 1

CLARK Genus	CLARK reads	SMART				BLAST			
		Mammalian	Same Genus	Other Genus	Unknown	Mammalian	Same Genus	Other Genus	Unknown
Top 10 classified									
*Alteromonas*	1,722	1,400	300	0	22	1,283	201	36	202
*Mycoplasma*	637	637	0	0	0	386	2	0	249
*Propionibacterium*	366	94	1	268	3	10	0	353	3
*Pandoravirus dulcis*	337	337	0	0	0	82	0	0	255
*Pandoravirus salinus*	275	273	0	0	2	89	0	0	186
*Bracovirus*	228	226	0	0	2	117	0	0	111
*Ichnovirus*	163	162	0	0	1	34	0	0	129
*Yersinia*	162	161	0	0	1	15	0	0	147
*Pseudomonas*	128	8	0	117	3	5	0	103	20
*Hepacivirus*	128	128	0	0	0	84	0	0	44

**Fig. 5:**
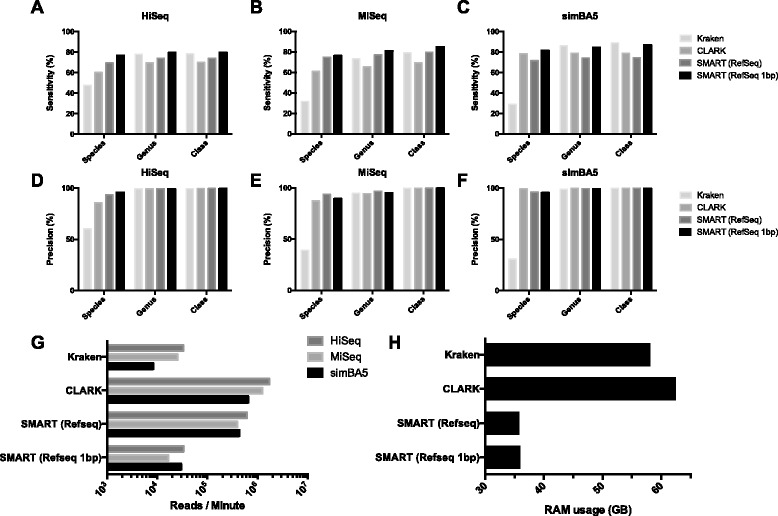
Comparison of accuracy, throughput, and memory utilization among Kraken, CLARK, and SMART built from the same RefSeq sequences. **a**, **b**, **c** Sensitivity at the level of species, genus, and class for simulated datasets (HiSeq, MiSeq, and simBA5). **d**, **e**, **f** Precision at the level of species, genus, and class. Throughput (**g**) and memory utilization (**h**) of datasets with 16 parallel threads in the same computing environment

When comparing SMART to Kraken and CLARK directly, a separate database for SMART was developed with all the bacterial, viral, and archaeal sequences from RefSeq. A total of 11,061 sequences were indexed by each tool. During execution each tool utilized all 16 CPUs for multithreading. Sensitivity, precision, throughput, and memory utilization are shown in Fig. 5. SMART utilized on average 2.24 GB of RAM per search program. Disk space of databases for Kraken, CLARK, and SMART were 151 GB, 113 GB, and 29 GB respectively.

## Discussion

By indexing every 30-mer in the NCBI GenBank with a multiplexed, parallel searching strategy, we were able to achieve an unsurpassed ability to classify reads against all currently catalogued DNA simultaneously while maintaining similar throughput, sensitivity and precision to Kraken and CLARK on simulated datasets. To the authors’ knowledge, this is the first metagenomic classification algorithm capable of efficiently matching against all the species and sequences present in the NCBI GenBank, allowing for a single step classification of microorganisms as well as large plant, mammalian, or invertebrate genomes from which the metagenomic sample may have been derived and allows for identification of novel sequences without pre- or post- filtering steps.

Kraken represented an improvement in throughput and accuracy in classification algorithms when released in 2014 [5]. During the construction of the Kraken-GB database, Wood et al. noted that there were several draft genomes that had included mislabeled DNA or included adapter sequences and cautioned against the interpretation of Kraken’s matches [5]. Our approach of searching the entire GenBank genomic DNA catalogue would protect against these false-positive matches as erroneous sequences would be present in multiple organisms and these results would label the read as ambiguous. However, this highlights the limitation and potential biases introduced by selective over-representation of certain species in the NCBI GenBank. For example, many of the animal models used in the biomedical science are overrepresented in the genomic DNA catalogued, as scientists are most interested in these organisms (Table 2). Hence false-positive matching may occur against these organisms if the true organism has not been sequenced yet. Statistical modeling could be used to generate matching likelihoods to each organism based on relative database representation.

With integration into Illumina BaseSpace, Kraken has rapidly become the bioinformatics pipeline used to analyze metagenomics next generation sequencing results. However, SMART has a number of advantages over Kraken. SMART employs a scalable infrastructure that is not dependent on a common database and can distribute the workload across many computational nodes. In addition, many metagenomics samples come from host-rich environments and Kraken suffers from false positive identification of microbial organisms. In our study, when comparing the human gut microbiome samples, Kraken could not classify 68.2 % of the reads compared to 57.6 % with our search strategy. With the conjunctival samples, Kraken identified numerous reads matching Mycoplasma, Pandoravirus dulcis, Pandoravirus salinus, and Human endogenous Retrovirus K113. By SMART and BLAST, all of these reads were of mammalian origin (Table 4).

In a direct comparison among Kraken, CLARK, and SMART using the same training RefSeq sequences and the same computing environment, CLARK and SMART were noted to have higher sensitivity and precision compared to Kraken at the species classification level. Without 1 basepair permutations, SMART was noted to have similar throughput to CLARK and with 1 basepair permutations, SMART was noted to have similar throughput to Kraken. SMART was noted to use significantly lower RAM compared to Kraken and CLARK. The main advantage of SMART appears to be utilizing a many-shard database approach to achieve horizontal scaling of a very large training set. While Kraken and CLARK have similar throughput and accuracy, they are unable to index a large training set that includes many mammalian, plant, fungal, and other protozoan organisms, both in the database construction phase and in analysis due to limitations in RAM. Since the sharded database can be loaded asynchronously in pieces, SMART can work in limited RAM environments without any changes to the algorithm by lowering the number of threads.

The exact k-mer matching approach has been used in several prior classification algorithms. SMART is similar to Kraken, CLARK, and LMAT in using exact k-mer matching for classification. However, SMART utilizes substantially lower RAM usage in the database construction phase by avoiding linking k-mers to a taxonomy tree and determining LCA. Unlike CLARK, SMART keeps all k-mers in the database and does not limit the search space by only keeping discriminatory k-mers. By using a deterministic sharding scheme, SMART is able to handle the expanded search space by asynchronously loading shards of database and allows for scalability. While the matching approach is similar to prior algorithms, SMART scales efficiently in a many-CPU, many-node environment and allows for accessing the entire NCBI GenBank in a single classification step.

Despite filtering human sequences in the conjunctival sample using BaseSpace prior to classification, Kraken (Table 4) and CLARK (Table 5) had many reads classified as bacterial or viral which were classified as mammalian by SMART. BLAST verified that the majority of these sequences were indeed mammalian. If an improved filtering step were implemented, or if Kraken or CLARK included mammalian genomes in their databases, their performance in host-rich metagenomics samples would have likely been improved. Unfortunately due to memory constraints on the database construction steps of both Kraken and CLARK, it was not possible for us to construct a database to include mammalian genomes in the evaluation databases for Kraken and CLARK. Inclusion of human and mammalian sequence filtering as an intrinsic component of the SMART protocol resulted in higher specificity of sequences assigned to non-host sources.

As the number of species sequenced grows, the NCBI GenBank will continue to expand, and the database shards used in this approach will also grow and consume more memory. At a certain point in the future each shard may consume too much memory and the database may need to be split with larger barcodes. However, computational infrastructure have also been growing in accordance to Moore’s law [32] and with cheaper costs in computer memory, this tipping point may be further away.

While we only benchmarked this approach in a cluster-computing environment, this deep search technique could be easily translated to a cloud computing infrastructure [33, 34]. These on-demand high-memory instances could be elastically created in parallel to handle each workload and destroyed after their use, allowing another layer of parallelization to occur. One limitation of the UW Hyak computing cluster that we faced was the relatively slow Input and Output (IO) performance of the network filesystem. In contrast, many of the cloud computing infrastructures are optimized for IO performance and this approach may benefit from implementation and tuning in a cloud environment.

Further improvements in this approach are possible to increase the throughput. In particular, the generation of 1 basepair permutations of the query may benefit from further optimization and from another MapReduce step. In addition, higher throughput would be achieved with the recruitment of more computational nodes in the cluster. This approach would also be applicable to RNASeq data in identifying gene transcripts and pathogen RNA by using a similar approach to index all the cDNA data in the NCBI GenBank. In particular viral transcripts may be proportionally enriched both in the GenBank catalogue as well as in the biological samples.

## Conclusions

We present the first scalable complete search approach to effectively classify metagenomics sequencing data using both exact 30-mer matching and 1 basepair permutations using the entirety of the NCBI GenBank. We anticipate this approach will be useful in identifying pathogens, characterizing complex microbiomes, and be extendable into labeling transcripts in RNASeq data.
